# Socrates in the fMRI Scanner: The Neurofoundations of Morality and the Challenge to Ethics

**DOI:** 10.1017/S0963180121000074

**Published:** 2021-10

**Authors:** Jon Rueda

**Affiliations:** University of Granada, Granada, Spain.

**Keywords:** human morality, moral behavior, neuroethics, neuroscience of ethics, normative ethics

## Abstract

The neuroscience of ethics is allegedly having a double impact. First, it is transforming the view of human morality through the discovery of the neurobiological underpinnings that influence moral behavior. Second, some neuroscientific findings are radically challenging traditional views on normative ethics. Both claims have some truth but are also overstated. In this article, the author shows that they can be understood together, although with different caveats, under the label of “neurofoundationalism.” Whereas the neuroscientific picture of human morality is undoubtedly valuable if we avoid neuroessentialistic portraits, the empirical disruption of normative ethics seems less plausible. The neuroscience of morality, however, is providing relevant evidence that any empirically informed ethical theory needs to critically consider. Although neuroethics is not going to bridge the is–ought divide, it may establish certain facts that require us to rethink the way we achieve our ethical aspirations.

## Introduction

The origins of Western moral philosophy have an indelible birthmark—*know thyself.* Following that Delphic precept, Socrates established an intimate relationship between self-knowledge and the pursuit of goodness.^1^ To behave well, you have to first know who you are. The discovery of the true self (i.e., the soul) is the path to envisage what is right. Indeed, the Socratic “inner eye” is spiritualistic, mainly because it is the way to perceive the genuine essences of “Justice” or “Kindness.” Socratic ethics leads to that innermost quest to discover who we are in order to discern how we should act. There is an explanation for this view that today may seem certainly strange. Like most Greeks, Socrates endorsed a teleological understanding of human nature.^2^ Human beings are “preprogrammed” organisms that have a natural purpose, an intrinsic finality. We have, in other words, a kind of software that we need to decode if we want to function as best as possible. The foundations of a morally good life are thus ingrained in our very nature.

More than two millennia later, ethics is now a discipline that has been predominantly disengaged from that Socratic spiritualist vision. The last two centuries of progress in the life sciences have drawn a scientific picture of the human species and its evolutionary origin. Moreover, empirical studies of human morality are a growing trend that seeks to shed light on the biological and psychological basis of our moral conduct.^3^ Neuroscience is precisely one of the leading fields that is studying the very nature of morality.

The term *neuroethics* has a twofold meaning. Adina Roskies established a landmark distinction between the ethics of neuroscience and the neuroscience of ethics.^4^ The former refers to the ethical aspects involved in neuroscientific research practices or in using specific neurotechnologies. The latter refers to the neuroscientific studies that are addressing the neuropsychological correlates that underlie human morality. In this article, I will focus on this second version of neuroethics as the neuroscience of ethics.^5^ I will address two claims about a double disruptive impact that the neuroscience of morality is allegedly provoking:

*Claim 1*: Neuroscience is radically transforming the view of human morality.

*Claim 2*: Neuroscience is radically challenging traditional views in normative ethics.

Neuroscience, on the one hand, is impacting our self-conception as moral beings. The knowledge provided by neuroscience could become essential to self-understand and change (even by biomedical means) our moral behavior. In its most radical stances, to Socrates’ surprise, it is raising a sort of “neuroessentialism”—the view which claims that “our brains define who we are,” or what is the same, to investigate the self we need to investigate the brain.^6^ On the other hand, we may ask whether neuroscience is transforming ethics as a discipline itself. There is an ongoing philosophical debate about the extent to which the results of neuroscientific research can radically alter long-standing views of traditional ethical theories.^7^ ^,^^8^ ^,^^9^ ^,^^10^ ^,^^11^ ^,^^12^

My thesis is that both contentious claims, though different in character, are intertwined: both relate to what I will label as *neurofoundationalism.* Neurofoundationalism is the view that neuroscience contributes to the foundational understanding of human morality. According to this perspective, neuroscience can significantly illuminate the underlying neural substrates of moral abilities and judgments (baseline of Claim 1) and/or lead to infer particular consequences in the normative domain (baseline of Claim 2).^13^ Not surprisingly, Tommaso Bruni et al. stated that “neuromoral theories” can lead both to a *descriptive grounding* (raising scientific understanding of human morality) and to a *normative grounding* (resulting in claims pertaining to the prescriptive realm).^14^ In this article, I will clarify the basis, the scope, and the philosophical problems deriving from these two claims. In parallel, it will be shown that some conceptions of neuroethics are not too far from the Socratic quest for self-knowledge.

The structure of my argument proceeds as follows. In Section “Gadfly in the Lab: The Neuroscience of Morality,” I will summarize some evidence of the neurobiological basis of moral conduct, and argue that these descriptive claims are more valuable as long as they avoid the construction of a neuroessentialist image of human morality. In Section “The Neuroscientific Challenge to Ethics,” I will address whether those empirical findings may disrupt ethical views and what (if any) are its consequences in the normative domain, and argue that the radical transformation of ethics, as a prescriptive discipline itself, is not plausible. Finally, I will conclude that any empirically informed ethical theory should indeed critically appraise the neuroscientific knowledge of human morality.

## Gadfly in the Lab: The Neuroscience of Morality

Morality is a hallmark of humanity.^15^ Admittedly, moral capacities are not an exclusive characteristic of our species.^16^ Human morality, however, has a neurobiological basis that distinguishes us from other animals in various aspects—and makes us similar in others. In this section, I will briefly sketch a body of evidence that supports the neurobiological basis of human morality, and concentrate on brain damage cases, neuroimaging, neuromodulation, and evolutionary theory approaches.

First, *brain lesion cases* provide noteworthy evidence of the neural underpinnings of morality. The study of specific brain injuries has advanced knowledge about the neuroanatomical correlates of moral processes. The case of Phineas Gage is probably the most famous example of how traumatic damage in a concrete brain region can considerably affect moral behavior. Phineas Gage was a railroad worker—and a person of “temperate habits”—who survived an accidental explosion in which an iron struck his head, breaking consequently the skull, passing through the anterior left lobe, and making its exit in the median line “breaking up considerable portions of brain.”^17^ As a result of the accident, Phineas Gage suffered irreversible damage in the ventromedial prefrontal cortex which impaired his social and moral behavior. After recovery, he was impaired in relation to ordinary decisionmaking skills, emotional abilities, anticipatory planning, and the willingness to respect sociomoral conventions, although he maintained the basic cognitive capacities and social knowledge.^18^ Beyond this example, nontraumatic disorders—such as neurodegenerative diseases or frontotemporal dementia—can also damage specific brain regions associated with different moral aptitudes.^19^ ^,^^20^

Second, *neuroimaging* provides scientific insight into the brain in action. A prominent neuroimaging technology is functional magnetic resonance imaging (fMRI), which since the nineties has had a great impact on public perception of neuroscience.^21^ ^,^^22^ This technique enables researchers to measure the neural activity of—healthy or unhealthy—participants during a specific moral task.^23^ More specifically, fMRI studies have been copious in investigating the neural substrates of moral judgment. When confronting diverse moral dilemmas, research subjects exhibit different brain activity that shows, for instance, if their response is predominantly emotional or predominantly cognitive.^24^ It helps, therefore, to theorize which are the salient characteristics of each experimental/control condition that might influence the corresponding neural correlate. Accordingly, the fMRI scanner can pave the way for showing “the hidden tectonics of the moral mind.”^25^ Other important neuroimaging techniques are computed tomography, electroencephalography, magnetic resonance imaging, magnetoencephalography, or positron emission tomography.

Third, *neurochemical modulation* and *brain stimulation* techniques can alter moral judgment and behavior. On the one hand, brain chemistry affects morality. Neuromodulators are brain chemicals that modify synaptic function, excitability, and neuronal dynamics.^26^ There are widely used pharmaceuticals—such as propranolol, selective serotonin reuptake inhibitors, or drugs that affect oxytocin—that modify moral dispositions.^27^ On the other hand, brain stimulation techniques—such as transcranial direct current stimulation, transcranial magnetic stimulation, and deep brain stimulation—can also modulate moral behavior through altering judgment, decisionmaking, prosocial dispositions, or aggressiveness.^28^ Basically, neurostimulation techniques apply electrodes (by invasive or noninvasive means) that boost specific bioelectrical currents of the brain.^29^ Furthermore, neurochemical modulation and neurostimulation can be used beyond treatment or prevention of diseases or health purposes, aiming to moral neuroenhancement.^30^ Moral enhancement is a daunting but exciting philosophical and scientific debate.^31^ ^,^^32^ Moral neuroenhancement consists, roughly, in a deliberate improvement of moral capacities and behavior beyond normal state by neurobiological and neurotechnological means.

Fourth, the building blocks of human morality have an *evolutionary origin.* It is broadly accepted that some (hardwired or at least prewired) features of human moral psychology evolved in the Pleistocene: they are an adaptation to the ancestral environment of hunter-gatherers in which human foragers live in highly interdependent small communities.^33^ ^,^^34^ ^,^^35^ In that sense, our evolutionarily forged moral psychology indicates an obvious mismatch between the lifestyle of our ancestors and the ethical challenges of today’s societies.^36^ Furthermore, humans share similarities in some moral dispositions (e.g., empathy) with their great ape relatives^37^ and also with other species of their primate lineage.^38^ ^,^^39^ Of course, some controversies remain an open debate, such as whether evolution could explain not only the innate architecture of particular moral capacities in humans, but also the specific content of widespread normative codes—as some nativist theories signal about the evolved foundations of morality.^40^ ^,^^41^

Those four strategies, if charitably interpreted, might constitute a valuable contribution to the understanding of human moral nature. This neuroscientific picture is not, however, without implications. Through this “process of self-discovery,” as we start “to understand ourselves better—who we are, and why we are the way we are—we will inevitably change ourselves in the process.”^42^ Moreover, neuroscience implies “the construction of a metaphysical mirror that will allow us to see ourselves for what we are and, perhaps, change our ways for the better.”^43^ After all, neuroethics is not too far off the Socratic aspiration. In the words of Kushner and Giordano: “To paraphrase Socrates, to know where one is going, it is best to recognize both where one is, and from whence one has come.”^44^

Thus, *Claim 1*—neuroscience is radically transforming the view of human morality—seems plausible. Yet, any neuroessentialistic depiction of human morality should be avoided. Neuroscience runs the risk of committing a hasty reduction if it equates the foundations of the moral phenomena merely to the brain. Although it “can be tempting to interpret neuroscience as the holy grail of self-knowledge so precious to ethics,”^45^ the explanatory power of neurosciences is often limited. First, although ethical reasoning is embodied in the brain and moral cognition has its neural constituents,^46^ there is no such “moral center” that can be located in only one place of the brain.^47^ Second, neural networks have a great plasticity that is modulated by social circumstances—nature via nature—in which the institutional environment influences the “neural ecology.”^48^ Third, the neuroimaging studies of hypothetical dilemmas are not the best manner to study daily life morality, because these moral judgment experiments have limited ecological validity.^49^ ^,^^50^ Fourth, culturally well-established moral patterns such as inclusivity show that humans can modify their evolved moral limitations, “even when doing so is not only fitness enhancing but even fitness reducing.”^51^ A finer-grained portrayal of human morality, therefore, cannot not rely only in the neuroscientific perspective.

## The Neuroscientific Challenge to Ethics

The fact that human morality has a neurobiological basis has opened stimulating debates. Some authors have interpreted neuroethics as a philosophical challenge;^52^ ^,^^53^ others have discussed whether neuroethics could constitute *new* ethics.^54^ ^,^^55^ ^,^^56^ ^,^^57^ What could establish such *novelty* is of course disputed. Michael Gazzaniga, for instance, ventured to claim that neuroethics gives the opportunity to develop a “brain-based ethics.”^58^ Conversely, Alasdair Macintyre pointed out that the study of the brain could jeopardize the perspective of ethics itself.^59^ ^,^^60^ In this section, I will concisely address three purported disruptive potential of neuroscience concerning normative ethics: conceptual amendments, debunking moral judgments, and bridging the is–ought divide.

First, the neuroscientific turn can lead to some considerable conceptual disruptions—major transformations in the way we conceive something. In relation to ethical theory, there are remarkable examples of concepts that are being revised in the light of recent brain science evidence.^61^ For instance, ancient philosophical disputes about free will have been reinvigorated through neuroscientific research.^62^ ^,^^63^ ^,^^64^ Furthermore, the philosophical discussion of topics such as moral agency is increasingly requiring the consideration of biological and psychological knowledge, when in the past the lack of empirical information about it was not considered inadequate.^65^ Other (nonexhaustive list of) concepts that are progressively being enriched by neuropsychological scrutiny include culpability, empathy, intention, intuition, punishment, rationality, responsibility, or self-control. The resulting changes in these concepts, in turn, may impact on other traditional ethical issues such as the omission/act distinction or the doctrine of double effect.^66^

Second, a prominent line of neuroscientific research has approached the neuropsychological underpinnings of moral judgments *characteristically* elicited by ethical theories like deontology or utilitarianism. Joshua Greene et al., for instance, showed that archetypical deontological responses to the footbridge version of the trolley dilemma were predominantly emotional.^67^ This apparently contradicts the foundations of deontological theories such as Kant’s based on rationality.^68^ In fact, it is now broadly agreed that intuitive emotional responses—whose relevance was diminished by former cognitive developmental theories—play a protagonist role in moral judgment.^69^ ^,^^70^ ^,^^71^ In this sense, the evidence provided by neuroimaging can be “informative” or “even revelatory” and, moreover, can revive old disputes between rationalists and emotivists sharpening new arguments.^72^ ^,^^73^ On the other hand, neurocognitive sciences have shown that morally irrelevant psychological factors can influence moral judgment which may raise doubt about its normative reliability.^74^ ^,^^75^ Furthermore, personality traits also influence the responses to characteristically utilitarian or deontological judgments.^76^ ^,^^77^ ^,^^78^

Third, we shall consider whether the neuroscience of morality is narrowing the is–ought abyss. Neuroethics is shedding light on how value judgments are rooted in biology and is telling “what brains do value.”^79^ It seems that, at least from a naturalistic framework in brain research, the kingdom of facts and the kingdom of norms are not so independent from each other. However, *à la Moore*, the need for avoiding the naturalistic fallacy—namely, inferring moral properties from any natural set of facts—is commonly pointed out.^80^ But once that peril has been averted, we shall acknowledge the *descriptive masquerade* of neuroethics. As Kathinka Evers et al. put forward:

although the “neuroscience of ethics” is typically considered descriptive, it has long been prescriptive: implicit assumptions about brain facts, their value, and their normative weight underlie the claim that neuroscientific findings will lead us to revise particular metaphysical and ethical notions.^81^

In my view, to admit the not purely descriptive function of the neuroscience of morality is not truly problematic for ethics. The longstanding Humean admonition of the invalidity of inferring a normative statement (i.e., an “ought”) from a purely descriptive one (i.e., a “fact”) is commonly evoked. Ironically, the is–ought divide is becoming the ultimate self-defense trench for moral philosophers in the face of the advance of the neuroscience of morality. Surely, from empirical findings of neuroscience on their own any substantive normative conclusion can barely be inferred, unless these findings are convoyed with other epistemic and moral premises.^82^ It has been said, therefore, that major neuroscientific findings have little normative significance to ethics.^83^ ^,^^84^ For sure, this is not to say that they do not have any normative significance at all. Thus, if from a neuroscientific finding by itself we cannot reach a normative conclusion, the case that relevant neuroempirical evidence can dramatically challenge any substantial ethical commitment seems fairly implausible. For an ethical theory to be completely discredited, not only do we need to know how the *de facto* brain mechanisms of morality work, but above all, we need normative ethical reasons for what we should do.

Consequently, *Claim 2*—the neuroscientific disruption of traditional ethical views—is mostly ungrounded and should be dismissed. In short, the prescriptive nature of ethics will not be radically challenged by the neuroscience of morality.

## Concluding Remarks: Toward a Neuroempirically Informed Ethical Theory

Ethics should not ignore neuroscientific progress. Current knowledge about the cerebral basis of human moral psychology is probably a small part of what we will come to know in the coming decades. But even if scientific understanding about the brain basis of human morality increases considerably, the manner ethical theories are developed does not need to dramatically change. In this article, I have addressed two claims related to what I have labeled as *neurofoundationalism*—the descriptive and normative grounding of ethics from neuromoral theories. I shall recapitulate my conclusions regarding both claims. First, although neuroscience is providing insightful evidence about the neural underpinnings of moral behavior, it does not have to lead us to an essentialist depiction of human morality. Second, neuroscience alone cannot radically change the foundations of traditional ethical theories. Whereas it is true that many ethical concepts, the way we understand moral judgements, and the relationship between facts and values may be affected by the development of neuroscience, the complete disruption of normative positions in moral philosophy is highly doubtful.

That said, there are at least two lessons to be learned from the neuroscience of morality. On the one hand, we may not be able to directly infer values from facts, but facts can make us rethink how we want to realize the values to which we aspire. A neuroempirically informed ethical theory could be thus more realistic, avoiding overdemanding “moral saints,” without renouncing the ideals of the normative domain. One valuable way in which neuroethics can contribute to the realization of normative goals is by providing insight into the myriad of ethically irrelevant neuropsychological factors that condition us in our daily moral lives. On the other hand, neuroscience does not compete against moral philosophy in the understanding of human morality. The relationship can be mutually beneficial if there is an attentive and thoughtful exchange regarding the contributions of both fields toward reformulating long-standing questions and providing novel answers. After all, “neuroethics is in some ways old wine in a new bottle.”^85^

Finally, the everlasting aspiration to self-knowledge is not only a birthmark of ethics, but it is also part and parcel of neuroethical research. However, the partial view of “fMRI myopia”^86^ in neuroethics might be as shortsighted as the essentialist Socratic inner eye. Neuroscience needs to be complemented with the perspective of other disciplines. Despite the Socratic aspiration of neuroethics, neuroscience need not be conceived as the Gadfly of our times that disseminates disturbing truths. Ethics should welcome and critically scrutinize the neuroscience of morality.

## Notes

1. Rappe SL. Socrates and self-knowledge. *APEIRON. A Journal for Ancient Philosophy and Science* 1995;**28**(1):1–24.

2.

Sedley D . Socrates, Darwin, and teleology. In: Rocca J , ed. Teleology in the Ancient World: Philosophical and Medical Approaches. Cambridge: CUP; 2017:23–4.

3.

Dworazik N , Rusch H . A brief history of experimental ethics. In: Luetge C , Rusch H , Uhl M , eds. Experimental Ethics. Toward an Empirical Moral Philosophy. Cham: Springer; 2014:38–56 .

4.

Roskies A . Neuroethics for the new millenium. Neuron 2002;35(1):21–31212360510.1016/s0896-6273(02)00763-8.

5. “Neuroscience of morality” is a more accurate expression. This is because it does not consist in the neuroscientific study of ethics (the philosophical discipline that deals with morality) as a discipline *per se*, but in the behavioral, psychological, and brain-based studies of different dimensions of human morality (such as moral judgments, cognition, reasoning, intuitions, emotions, and so on).

6. See note 4, Roskies 2002, at 22.

7.

Berker S . The normative insignificance of neuroscience. Philosophy and Public Affairs 2009;37(4):293–329 .

8.

Levy N . Neuroethics: A new way of doing ethics. AJOB Neuroscience 2011;2(2):3–9 2231897610.1080/21507740.2011.557683PMC3272467.

9.

Greene JD . Beyond point-and-shoot morality: Why cognitive (neuro)science matters for ethics. Ethics 2014;124(4):695–726 .

10. Racine E, Dubljević V, Jox RJ, Baertschi B, Christensen JF, Farisco M, et al. Can neuroscience contribute to practical ethics? A critical review and discussion of the methodological and translational challenges of the neuroscience of ethics. *Bioethics* 2017;**31**(5):328–37.

11.

Buller T . The new ethics of neuroethics. Cambridge Quarterly of Healthcare Ethics 2018;27(4):558–653072041410.1017/S0963180118000087.

12.

Racine E , Sample M . Two problematic foundations of neuroethics and pragmatist reconstructions. Cambridge Quarterly of Healthcare Ethics 2018;27(4):566–773072041210.1017/S0963180118000099.

13. Note that one who endorses Claim 1 does not need to endorse Claim 2, but one who endorses Claim 2 needs to grant validity to Claim 1.

14.

Bruni T , Mameli M , Rini RA . The science of morality and its normative implications. Neuroethics 2014;7(2):159–72.

15.

Ayala FJ . The difference of being human: Morality. Proceedings of the National Academy of Sciences of the United States of America 2010;107(2 Suppl):9015–222044509110.1073/pnas.0914616107PMC3024030.

16.

Monsó S , Benz-Schwarzburg J , Bremhorst A . Animal morality: What it means and why it matters. Journal of Ethics 2018;22(3–4):283–310 10.1007/s10892-018-9275-3PMC640464230930677.

17.

Harlow JM . Passage of an iron rod through the head. The Boston Medical and Surgical Journal 1848;39:389–93PMC990462637319500.

18.

Damasio AR . Descartes’ Error: Emotion, Reason, and the Human Brain. New York: G.P. Putnam; 1994 .

19.

Mendez MF , Anderson E , Shapira JS . An investigation of moral judgement in frontotemporal dementia. Cognitive and Behavioral Neurology 2005;18(4):193–71634039110.1097/01.wnn.0000191292.17964.bb.

20.

Mendez MF . The neurobiology of moral behavior: Review and neuropsychiatric implications. CNS Spectrums 2009;14(11):608–202017368610.1017/s1092852900023853PMC3163302.

21.

Illes J . Neuroethics in a new era of neuroimaging. American Journal of Neuroradiology 2003;24(9):1739–4114561594PMC7976301.

22.

Racine E , Bar-Ilan O, Illes J. fMRI in the public eye. Nature Reviews Neuroscience 2005;6(2):159–641568522110.1038/nrn1609PMC1524852.

23. Prehn K, Heekeren H. Moral brains—Possibilities and limits of the neuroscience of ethics. In: Christen M, van Schaik C, Fischer J, Huppenbauer M, Tanner C, eds. *Empirically Informed Ethics: Morality between Facts and Norms.* Cham: Springer; 2014:137–57, at 143.

24.

Greene JD , Sommerville RB , Nystrom LE , Darley JM , Cohen JD . An fMRI investigation of emotional engagement in moral judgment. Science 2001;293(5537):2105–81155789510.1126/science.1062872.

25.

Greene JD . Beyond point-and-shoot morality: Why cognitive (neuro)science matters for ethics. Ethics 2014;124(4):695–726, at 705.

26.

Crockett MJ . Morphing morals: Neurochemical modulation of moral judgment and behavior. In: Liao MS , ed. Moral Brains: The Neuroscience of Morality. New York: OUP; 2016:237–45, at 237.

27.

Levy N , Douglas T , Kahane G , Terbeck S , Cowen PJ , Hewstone M , et al. Are you morally modified? The moral effects of widely used pharmaceuticals. Philosophy, Psychiatry & Psychology 2014;21(2):111–2510.1353/ppp.2014.0023PMC439897925892904.

28.

Di Nuzzo C , Ferrucci R , Gianoli E , Reitano M , Tedino D , Ruggiero F , et al. How brain stimulation techniques can affect moral and social behaviour. Journal of Cognitive Enhancement 2018;2(4):335–47.

29.

Bourzac K . Neurostimulation: Bright sparks. Nature 2016;531(7592):S6–S8 2693452510.1038/531S6a.

30. Earp BD, Douglas T, Savulescu J. Moral neuroenhancement. In: Johnson LSM, Rommelfanger KS, eds. *The Routledge Handbook of Neuroethics.* New York: Routledge; 2018:166–84.

31.

Rueda J . Climate change, moral bioenhancement and the ultimate mostropic. Ramon Llull Journal of Applied Ethics 2020;11:277–303 .

32. Rueda J, Lara F. Virtual reality and empathy enhancement: Ethical aspects. *Frontiers in Robotics and AI* 2020;7(November):506984.

33. van Schaik C, Burkart JM, Jaeggi AV, von Rohr CR. Morality as a biological adaptation—An evolutionary model based on the lifestyle of human foragers. In: Christen M, van Schaik C, Fischer J, Huppenbauer M, Tanner C, eds. *Empirically Informed Ethics: Morality between Facts and Norms.* Cham: Springer; 2014:65–84.

34.

Burkart JM , Brügger RK , van Schaik CP . Evolutionary origins of morality : Insights from non-human primates. Frontiers in Sociology 2018;3(July):1–12 .

35.

Tomasello M . Precís of a natural history of human morality. Philosophical Psychology 2018;31(5):661–8.

36.

Persson I , Savulescu, J. Unfit for the Future: The Need for Moral Enhancement, Oxford: OUP; 2012 .

37. de Waal FBM. The antiquity of empathy. *Science* 2012;**336**(6083):874–6.

38.

Brosnan, SF . Precursors of morality—Evidence for moral behaviors in non-human primates. In: Christen M , van Schaik C , Fischer J , Huppenbauer M , Tanner C , eds. Empirically Informed Ethics: Morality between Facts and Norms. Cham: Springer; 2014:85–98 .

39. See note 34, Burkart et al. 2018.

40. Haidt J, Joseph C. The moral mind: How five sets of innate intuitions guide the development of many culture-specific virtues, and perhaps even modules. In: Carruthers P, Laurence S, Stich S, eds. *The Innate Mind.* Vol. 3. New York: OUP; 2007:367–91.

41. See also Mikhail J. Universal moral grammar: Theory, evidence and the future. *Trends in Cognitive Sciences* 2007;**11**(4):143–52.

42.

Greene J . From neural “is” to moral “ought”: What are the moral implications of neuroscientific moral psychology? Nature Reviews Neuroscience 2003;4(10):846–50, at 8501452338410.1038/nrn1224.

43.

Greene JD . Social neuroscience and the soul’s last stand. In: Todorov A , Fiske S , Prentics D , eds. Social Neuroscience: Toward Understanding the Underpinnings of the Social Mind. Oxford: OUP; 2006:263–73, at 272.

44.

Kushner T , Giordano J . Neuroethics: Cashing the reality check. Cambridge Quarterly of Healthcare Ethics 2017;26(4):524–5, at 5252893733610.1017/S0963180117000056.

45. See note 12, Racine, Sample 2018, at 570.

46.

Casebeer WD . Moral cognition and its neural constituents. Nature Reviews Neuroscience 2003;4(10):840–61452338310.1038/nrn1223.

47. See note 23, Prehn, Heekeren 2014, at 156.

48.

Avram M , Giordano J . Neuroethics: Some things old, some things new, some things borrowed … and to do. AJOB Neuroscience 2014;5(4):23–5, at 24.

49. Alfano M. Book review: Liao SM, ed. Moral brains: The neuroscience of morality. *Ethical Theory and Moral Practice* 2017;**20**(3):671–4.

50. The opposite via of extrapolation—from the study of ordinary moral situations to deeply conflictive dilemmas—is also problematic. See Kahane G. Is, ought, and the brain. In: Liao, M. ed. *Moral Brains: The Neuroscience of Morality.* New York: OUP; 2016:281–311, at 306n42.

51. Buchanan A, Powell R. The limits of evolutionary explanations of morality and their implications for moral progress. *Ethics* 2015;**126**(1):37–67, at 63–4.

52. Evers K. Neuroethics: A philosophical challenge. *American Journal of Bioethics* 2005;5(2):31–3.

53.

Churchland PS . The impact of neuroscience on philosophy. Neuron 2008;60(3):409–111899581310.1016/j.neuron.2008.10.023.

54.

Knoppers BM . Neuroethics, new ethics? American Journal of Bioethics 2005;5(2):33 10.1080/1526516059096039316036697.

55. Cabrera L. Neuroethics: A new way to do ethics or a new understanding of ethics? *AJOB Neuroscience* 2011;**2**(2):25–6.

56. See note 48, Avram, Giordano 2014, at 23–5.

57. Buller T. The new ethics of neuroethics. *Cambridge Quarterly of Healthcare Ethics* 2018;27(4):558–65.

58. Gazzaniga MS. *The Ethical Brain.* New York: Dana Press; 2005.

59. MacIntyre A. What can moral philosophers learn from the study of the brain? *Philosophy and Phenomenological Research* 1998;**58**(4):865–6.

60. See note 12, Racine, Sample 2018, at 569.

61. See note 10, Racine et al. 2017, at 331.

62. Libet B. Do we have free will? *Journal of Consciousness Studies* 1999;**6**(8–9):47–57.

63.

Moreno JD . Neuroethics: An agenda for neuroscience and society. Nature Reviews Neuroscience 2003;4(2):149–531256328610.1038/nrn1031.

64.

Häyry M . Neuroethical theories. Cambridge Quarterly of Healthcare Ethics 2010;19(2):165–782022610010.1017/S0963180109990430PMC3605912.

65. Evers K, Salles A, Farisco M. Theoretical framing of neuroethics: The need for a conceptual approach. In: Racine E, Aspler J, eds. *Debates About Neuroethics. Perspectives on Its Development, Focus, and Future.* Cham: Springer; 2017:89–107, at 91.

66. See note 8, Levy 2011.

67. See note 24, Greene et al. 2001.

68. Greene JD. The secret joke of Kant’s soul*.* In: Sinnott-Armstrong W, ed. *Moral Psychology, Vol. 3. The Neuroscience of Morality: Emotion, Brain Disorders, and Development.* Cambridge, MA: MIT Press; 2008:35–80.

69. See note 18, Damasio 1994.

70. See note 24, Green et al. 2001.

71.

Haidt J . The emotional dog and its rational tail: A social intuitionist approach to moral judgment. Psychological Review 2001;108(4):814–341169912010.1037/0033-295x.108.4.814.

72.

Prinz J . Sentimentalism and the moral brain. In: Liao, M . ed. Moral Brains: The Neuroscience of Morality. New York: OUP; 2016:45–73, at 69.

73. Some authors explicitly endorse particular ethical theories like consequentialism (see note 25, Greene 2014) or virtue ethics (see note 46, Casebeer 2003; and see note 40, Haidt, Joseph 2007). Needless to say, this is not problematic *per se.* Still, none of these authors completely disassociate their normative convictions from the neuroscientific evidence with which they engage. This can be especially problematic if the study of the prototypical judgments of some normative theories is based on simplified conceptions—at best—or strawman conceptions—at worst. Thus, we should be suspicious of any attempt to discredit any particular ethical theory across the board on the sole basis of neuroscientific evidence.

74. See note 7, Berker 2009.

75. Earp BD, Lewis J, Dranseika V, Hannikainen I. Experimental philosophical bioethics and normative inference. *Theoretical Medicine & Bioethics*; forthcoming.

76.

Bartels DM , Pizarro DA . The mismeasure of morals: Antisocial personality traits predict utilitarian responses to moral dilemmas. Cognition 2011;121(1):154–612175719110.1016/j.cognition.2011.05.010.

77.

Djeriouat H , Trémolière B. The dark triad of personality and utilitarian moral judgment: The mediating role of honesty/humility and harm/care. Personality and Individual Differences 2014;67:11–6.

78. Smillie LD, Katic M, Laham, SM. Personality and moral judgment: Curious consequentialists and polite deontologists. *Journal of Personality*, 2020 advance online publication; available at https://doi.org/10.1111/jopy.12598 (last accessed 17 Mar 2021).

79. See note 53, Churchland 2008, at 411n.

80. See note 42, Greene 2003.

81. See note 65, Evers et al. 2017, at 94.

82. See note 50, Kahane 2016.

83. See note 7, Berker 2009.

84. See note 50, Kahane 2016.

85. See note 63, Moreno 2003, at 153.

86. See note 49, Alfano 2017.

## Notes

Funding for open access charge: Universidad de Granada.

